# Tracking elusive cargo: Illuminating spatio‐temporal Type 3 effector protein dynamics using reporters

**DOI:** 10.1111/cmi.12797

**Published:** 2017-11-23

**Authors:** Nicky O'Boyle, James P. R. Connolly, Andrew J. Roe

**Affiliations:** ^1^ Institute of Infection, Immunity & Inflammation, College of Medical, Veterinary and Life Sciences University of Glasgow Glasgow UK

**Keywords:** effector, localisation, methods, protein, reporter, secretion

## Abstract

Type 3 secretion systems form an integral part of the arsenal of many pathogenic bacteria. These injection machines, together with their cargo of subversive effector proteins, are capable of manipulating the cellular environment of the host in order to ensure persistence of the pathogen. In order to fully appreciate the functions of Type 3 effectors, it is necessary to gain spatio‐temporal knowledge of each effector during the process of infection. A number of genetic modifications have been exploited in order to reveal effector protein secretion, translocation and subsequent activity, and localisation within host cells. In this review, we will discuss the many available approaches for tracking effector protein dynamics and discuss the challenges faced to improve the current technologies and gain a clearer picture of effector protein function.

## 1 INTRODUCTION

The successful proliferation of a bacterial species depends upon their ability to colonise an appropriate niche. Fitness within a specific setting can be brought about by sensing cues in the environment and subsequent modulation of colonisation and other key virulence factors (Connolly, Finlay, & Roe, 2015; Letchumanan et al., 2017; Mellies, Barron, & Carmona, 2007). Pathogenic bacteria have evolved diverse strategies to hijack host cellular processes thereby manipulating their environment and enabling persistence within the host. One such strategy involves the direct injection of effector proteins from the bacterial cytosol through a complex apparatus known as a Type 3 secretion systems (T3SS) into the host cell cytosol where they can exert their effect (Cornelis & Van Gijsegem, 2000; Dean & Kenny, 2009; Wong et al., 2011).

The T3SS is a 3.5 megadalton complex consisting of a number of bacterial membrane embedded components, a hollow proteinaceous needle, and a tip translocon apparatus, which becomes embedded in the host cell membrane thereby allowing the formation of a continuous conduit from the bacterial cytosol to the host cytosol (Marlovits et al., 2004; Radics, Königsmaier, & Marlovits, 2014). Effector proteins are recognised by the T3SS‐associated ATPase at the cytoplasmic membrane where their cognate chaperones are released, and they are unfolded to allow them to pass through the 20 Å T3SS channel in an ATP‐driven manner (Akeda & Galán, 2005). The mechanism of effector secretion and needle complex physiology are extremely important considerations when designing suitable effector‐reporter fusions for the study of effector translocation.

Effectors are secreted in a tightly regulated hierarchical manner and subvert diverse biological processes such as apoptosis, autophagy, inflammation, cytoskeletal remodelling, and membrane trafficking (Deng et al., 2017; O'Boyle & Boyd, 2014). Type 3 effectors often orchestrate complex signalling dynamics within the cell that establishes a delicate balance between colonisation and proliferation of the pathogen and modulation of host cell toxicity and defences. These dynamics underpin the infection strategies of many important bacterial pathogens including enterohemorrhagic Escherichia coli (EHEC), enteropathogenic E. coli (EPEC), *Shigella flexneri*, *Salmonella enterica*, *Yersinia spp*, *Pseudomonas spp* and Vibrio parahaemolyticus (Troisfontaines & Cornelis, 2005).

Classically, an understanding of protein function and localisation would be addressed by creating a fusion of the protein of interest to green fluorescent protein (GFP). However, the stable nature of the GFP β‐barrel and the size of the GFP “cylinder,” at around 24 Å in diameter, preclude secretion of effector‐GFP hybrid proteins (Akeda & Galán, 2005). GFP chromophores strictly require molecular oxygen for maturation of fluorescence and, as such, fluorescence may be inhibited in specific cellular microenvironments such as endocytic vacuoles and phagolysosomes (Hoffmann et al., 2010). An alternative strategy, which has been employed with varying degrees of success, is to transfect or microinject an appropriate cell line with a fluorescent protein‐effector fusion (Deslandes et al., 2003; Gawthorne et al., 2012; Yoshida et al., 2002). In this case, however, the concentration of effector protein in the host cell will inevitably be drastically different to that observed during infection. It is also important to consider that effector proteins are injected as a “suite” and in some cases, have antagonistic or synergistic roles (Chang et al., 2005; Van Engelenburg & Palmer, 2010). As a result, this highly reductionist approach can be prone to issues with improper localisation or functionality. This has led to intensive efforts in the development of innovative technologies to facilitate the monitoring of effector translocation through the T3SS and their subsequent localisation within the host cell.

**Figure 1:**
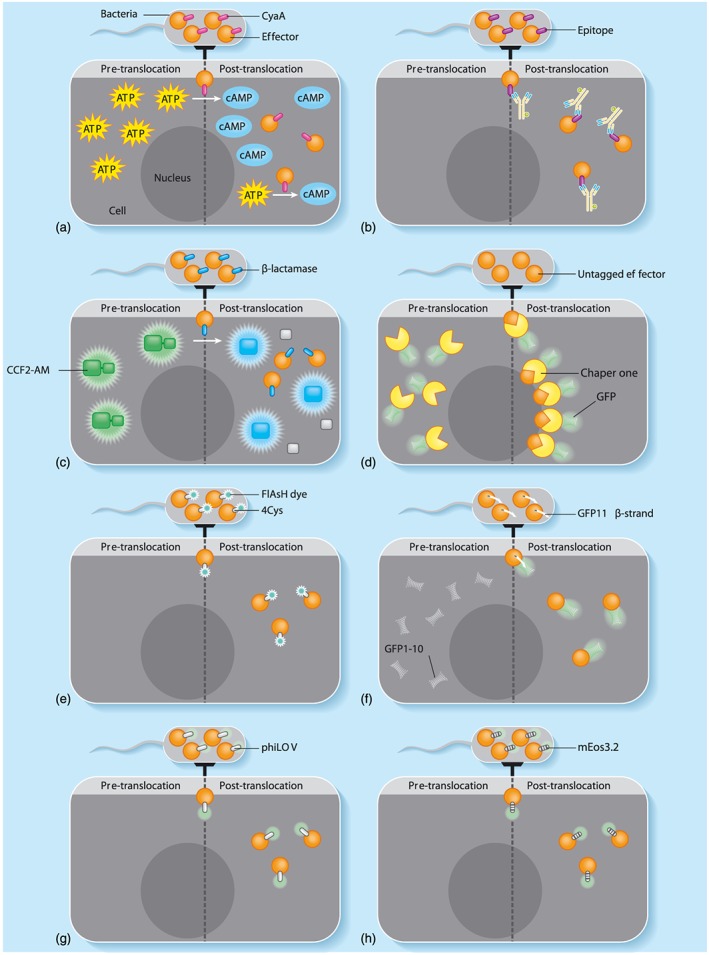
Schematic summary of the principle methodologies for tracking effector proteins during infection. (a) Bulk assessment of translocation via effector‐CyaA fusion (Section 2.1). Effector translocation results in host cell calmodulin‐dependent cAMP accumulation, which can be measured by enzyme‐linked immunosorbent assay. (b) Immunofluorescent detection of epitope‐tagged effectors in fixed tissues (Section 2.2). The translocated effector can be detected using commercially available antibodies with specificity for the chosen epitope tag. (c) Detection of TEM‐1 β‐lactamase‐effector fusions via spectral shifting of the fluorescence resonance energy transfer substrate coumarin cephalosporin fluorescein (CCF2‐AM; Section 2.3). Cleavage of the CCF2‐AM substrate shifts the emission spectrum of the fluorophore from green to blue allowing detection of translocation with high sensitivity. (d) Analysis of subcellular localisation of untagged effectors via recruitment of host‐expressed chaperone‐green fluorescent protein (GFP) fusion (Section 2.4). An un‐tagged effector protein can be visualised in the host cell upon binding to its fluorescently labelled chaperone when ectopically expressed via transfection. (e) Direct 4Cys‐FlAsH labelling of effectors (Section 2.5). Bacteria can be preloaded with FlAsH dye, which binds to the 4Cys tag and emits green fluorescence, thereby allowing real‐time tracking of effector injection. (f) Tracking of effectors within the host cells using split‐GFP fluorescence complementation (Section 2.6). Fusion of the GFP_11_ β‐strand to the incomplete/inactive host‐expressed GFP_1–10_, results in fluorescence complementation, allowing indirect assessment of translocation. (g) Direct labelling of effectors with phiLOV (Section 2.7). The phiLOV tag binds flavin mononucleotide, emitting fluorescence in the green spectrum, which allows direct analysis of effector secretion. (h) Direct labelling of effectors with photo‐switchable fluorescent protein such as mEos3.2 for super‐resolution microscopy (Section 2.8). Photo‐switchable fluorescent tags allow for reversible transition between on and off states that yields spatial resolution beyond the diffraction limit of light

In this review, we will discuss the methods that have facilitated an improved understanding of effector functionality (summarised in Figure 1). We will attempt to provide a historical perspective of the various reporter tools and provide a critical assessment of the benefits and limitations of each technique. It is worth noting that in the past decade, there have been many advances in light microscopy: optics, cameras, software, and super‐resolution techniques that have markedly improved the ability of researchers to study protein localisation. Issues such as phototoxicity and photobleaching, commonly associated with repeated exposure of fluorescently labelled cells during conventional laser scanning microscopy, can be reduced by platforms such as spinning disc confocal microscopy (Stehbens, Pemble, Murrow, & Wittmann, 2012). Further reductions in photodynamic damage can be obtained with multiphoton laser scanning microscopy, particularly where three‐dimensional real‐time imaging is required (Denk, Strickler, & Webb, 1990). Indeed, the successful application of nearly all the approaches detailed below is highly dependent on an optimal microscopy set‐up.

## 2 EFFECTOR‐TAGGING METHODOLOGIES

### 2.1 Enzymatic tags for detection of translocation

Prior to establishment of the T3SS model, it was known that under certain conditions, many pathogenic bacteria exported a subset of proteins. It was observed that when *Yersinia pseudotuberculosis* was cultured in medium containing low concentrations of calcium, several Yops (*Yersinia* outer proteins) were secreted into the supernatant and could be detected by SDS PAGE (Michiels, Wattiau, Brasseur, Ruysschaert, & Cornelis, 1990). *Yersinia* species were known to subvert phagocytosis and invade epithelial cells (Lian, Hwang, & Pai, 1987; Miller & Falkow, 1988); however, a specific mechanism whereby Yops could carry out these processes had not been demonstrated. One of the first methods used to determine whether effectors could be delivered into host cells was to fuse the protein to an enzyme or substrate that required a host‐derived counterpart for activity. Sory and Cornelis (1994) demonstrated that by creating a hybrid protein consisting of the *Bordetella pertussis* CyaA protein bound to the N‐terminus of YopE, translocation into the host cell could be detected and quantitated. The cyclase domain of CyaA induces accumulation of cAMP in the host cell, which can be easily quantitated by enzyme‐linked immunosorbent assay. Importantly, CyaA cyclase activity requires binding of the eukaryotic secondary messenger calmodulin, so no background cAMP is produced in the bacterial cell prior to translocation (Wolff, Cook, Goldhammer, & Berkowitz, 1980). The authors demonstrated that translocation of YopE occurred within 45 min of infection using HeLa cells and that translocation was dependent upon YadA‐mediated adhesion (Sory & Cornelis, 1994). The authors also demonstrated that strains carrying inactivating insertions in the *yopB/D* genes, which code for needle tip translocon components were unable to translocate YopE‐CyaA.

Translocation of *Yersinia pestis* Yops (YopE, YopH, LcrQ, YopK, YopN, and YopJ) has more recently been demonstrated using the small (13‐residue) glycogen synthase kinase (GSK) tag (Torruellas Garcia et al., 2006). Upon translocation, the tag is phosphorylated by unknown host cell kinases and phosphorylation can be detected by immuno‐blotting using a phospho‐specific GSK‐3β antibody. The reduced size of this tag was found to lower interference with effector secretion and translocation when compared with the 398‐amino acid CyaA fusion. Indeed GSK‐tagged YopE was found to display improved secretion and translocation compared to ELK‐tagged (a 35‐residue tag, which is also phosphorylated within the host cell) YopE (Torruellas Garcia et al., 2006).

The Cre recombinase has also been successfully employed as a tag to detect translocation of the *Salmonella* effector SopE into COS‐2 cells transfected with a GFP reporter plasmid. Translocation of SopE‐Cre during infection resulted in activation of GFP expression via recombination of *loxP* sites in the Cre reporter plasmid, which was measured by flow cytometry (Briones, Hofreuter, & Galán, 2006). This strategy had previously been employed to demonstrate translocation of the *Agrobacterium tumefaciens* Type IV secretion system effector Vir into transgenic *Arabidopsis* that possessed a similar Cre‐activated GFP expression construct (Vergunst et al., 2005). This importantly also demonstrated applicability of the system in vivo.

Although these enzymatic methods are useful for demonstrating translocation, quantitation is indirect and sample preparation usually requires collection of crude cell lysates at fixed time‐points, so no spatio‐temporal information is garnered. Experimental results are also obtained from the complete population of cells; some of which may not be infected, resulting in poor sensitivity levels. Although such assays are a useful means of demonstrating translocation, they are best served as complementary to microscopic methodologies that illustrate both translocation and localisation within single host cells.

### 2.2 Epitope tags for detection via immunofluorescence on fixed tissues

Epitope tags represent perhaps the most common means of detecting effector proteins microscopically. These are typically short peptides that have minimal effects on protein functionality. When fused either N‐ or more commonly C‐terminally to the protein of interest, they can be detected using commercially available antibodies thus revealing effector expression and localisation. Commonly used epitope tags include FLAG, HA, myc, T7, HSV, M45 and 3XFLAG with size ranges from 7 to 22 residues.

EPEC and EHEC use their T3SS to engage in intimate adherence with the surface of the intestinal epithelium (Kaper, Nataro, & Mobley, 2004). Epitope‐tagged variants of the translocated intimin receptor (Tir) protein revealed interesting aspects of its role in colonisation. Immunofluorescence of T7‐ and HSV‐tagged Tir revealed accumulation in actin‐rich regions of bacterial adhesion on the apical surface of infected HeLa cells (Kenny et al., 1997). Tir was also found to bind the bacterial adhesin intimin within these actin‐rich regions forming a protruding pedestal known as an attaching/effacing lesion. This was the first description of a pathogen directly injecting a receptor for its adhesin into the host cell.

*S. enterica* possesses two T3SSs; one of which (SPI1) is used predominantly to trigger actin rearrangements leading to cellular invasion, and the other (SPI2) is involved in maturation of the *Salmonella*‐containing vacuole (SCV) and dampening of host responses to allow intravacuolar replication (Haraga, Ohlson, & Miller, 2008). The accumulation of six SPI1 effector proteins with direct and indirect roles in cellular invasion via actin cytoskeleton reorganisation was elegantly analysed using indirect immunofluorescence on infected fibroblasts (Cain, Hayward, & Koronakis, 2004). Although antibodies could be raised against the immunogenic SipA and SipC, C‐terminal FLAG tags were employed for the detection of SopE, SopE2, SopB, and SptP. The effectors were found to localise to the cell periphery and to sites of bacterial attachment/invasion where membrane ruffling and actin reorganisation occurred. An interesting co‐operative function between the SPI1 secreted effector SipA and SPI2 effectors SifA and PipB2 was revealed using SipA^FLAG^, SifA^HA^ and PipB2^HA^ epitope‐tagged variants (Brawn, Hayward, & Koronakis, 2007). It was observed that SipA persisted after invasion and localised to the SCV where it was required for maximal intracellular replication and juxta‐nuclear positioning of the SCV. Positioning of the SCV was known to be regulated by the SPI2 effectors SifA and PipB2 that associate with dynein and kinesin‐associated tubules respectively. This interplay between SPI1 and SPI2 effectors shed new light on the spatial dynamics of intracellular replication of *Salmonella*.

Interestingly, the enzymatic reporters CyaA and GSK that were described in Section 2.1 also display antigenic properties, and these have been exploited for spatial analysis. The *Chlamydia trachomatis* fusion proteins IncD‐CyaA and IncD‐GSK were visualised by indirect immunofluorescence using anti‐CyaA and anti‐GSK antibodies respectively (Bauler & Hackstadt, 2014). The effector‐fusions were visualised both intrabacterially and within infected HeLa cells where they localised to the inclusion membrane.

The major limitation of labelling epitope‐tagged effector proteins is the fact that sample preparation requires fixation and permabilisation prior to effector detection. Such treatments can result in altered morphology and apparent protein localisation (Schnell, Dijk, Sjollema, & Giepmans, 2012). Fixation also precludes real time analysis. As real time methods become more commonplace, epitope tagging of effector proteins is still useful for confirming experimental observations. Perhaps, the greatest advantage of this approach is the inherent ability to amplify the signal from diffuse or poorly expressed effectors using secondary or tertiary antibody labelling. The wide variety of epitope tags also allows for greater flexibility in multiplex colocalisation analysis. In order to complement real time observations of the association between PipB2 and the anterograde microtubular motor protein kinesin, it was demonstrated that PipB2^3XFLAG^ also showed clear signal overlap with kinesin when expressed from a chromosomally integrated cassette (Van Engelenburg & Palmer, 2010). Importantly, this demonstrates that signal can easily be detected from epitope‐tagged chromosomal fusions that are under the control of native regulatory elements.

### 2.3 β‐lactamase fluorescence resonance energy transfer reporters

Translocation of effector proteins can also be determined microscopically using enzymatic tags that alter the fluorescence spectrum of a substrate in the host cell cytosol. This method involves fusion of TEM‐1 β‐lactamase to the effector of interest. The cells are stained with coumarin cephalosporin fluorescein (CCF2‐AM), which freely permeates the cell membrane and emits in the green light (520 nm) range. Cleavage of the CCF2‐AM fluorophore by the translocated effector‐TEM fusion results in an emission shift from green to blue (447 nm) fluorescence (Zlokarnik et al., 1998).

It was observed that EPEC expressing TEM‐1 fusions of the cytoplasmic proteins maltose binding protein and glutathione *S*‐transferase were unable to exert a fluorescence shift on infected HeLa cells, whereas the effector proteins Cif, Tir, Map, and EspF caused a shift from green to blue (Charpentier & Oswald, 2004). The authors also identified an exchangeable N‐terminal region of 20 amino acids that was required for secretion by identifying truncates of each effector protein which could no longer exert a fluorescence shift. A semiquantitative assessment of bulk translocation within an entire infected tissue culture well was provided by expressing emission ratio at 460 nm compared to that at 530 nm. This approach has effectively been scaled up and applied for real‐time quantitation of Tir, Map, EspF, EspG, EspH, and EspZ translocation (Mills, Baruch, Charpentier, Kobi, & Rosenshine, 2008). The effectors were shown to have differential rates of secretion depending on whether the effector fusions were expressed chromosomally or overexpressed on a plasmid. It was also apparent that effectors were translocated in a hierarchical manner as Tir concentration reached a steady state in the host cell at 40 min, while Map was undetected at this time‐point and required 60 min to reach a steady state.

Similarly, translocation of *S*. *enterica* flagellin (FliC) into macrophages was demonstrated by fluorescence resonance energy transfer (FRET) and was found to be dependent upon SPI1 but not the flagellar secretion apparatus (Sun, Rolán, & Tsolis, 2007). The authors also employed flow cytometry to assess the proportion of the macrophage population displaying a FRET shift at a given time post infection.

Advantages of the FRET system include high sensitivity with fewer than 100 molecules of TEM‐1 being sufficient for detection (Zlokarnik et al., 1998). However, although microscopic analysis of host cells containing translocated effectors is possible, the fluorescent signal is not confined to the effector protein's subcellular location as the fluorophore diffuses throughout the cell. It should also be noted that the rate of translocation recorded in real‐time studies is only semiquantitative as it depends upon the kinetics of enzymatic cleavage of the CCF2 fluorophore. CCF2 levels in the cell can become depleted after as little as 60 min of incubation (Mills et al., 2008), and as such, the time‐point at which the dye is added must be carefully considered depending on the effectors being studied.

### 2.4 Indirect detection via fluorescent chaperone binding

Interesting insight into the spatio‐temporal aspects of SPI1 effector protein functionality in cellular invasion has been obtained using fluorescent chaperones expressed in the host cell. This process involves transfection of a host cell with a well‐characterised Type 3 chaperone‐GFP fusion. When unmodified effector proteins are translocated into the host cell, they recruit the chaperone‐GFP fusion, thereby revealing the subcellular localisation of the effector. The *Salmonella* SPI1 effector SipA was shown to recruit the chaperone GFP‐InvB to a region of bacterial docking and actin remodelling (Schlumberger et al., 2005). The principal role of SPI1 is to induce cellular uptake of *Salmonella* via membrane ruffling and as such, wild type bacteria that secrete SPI1 effectors exhibit dynamic movement on the epithelial cell surface. To assess SipA translocation in real time, a *sopABEE2* deletion mutant was employed which lacked the ability to induce invasion or membrane ruffling but retained the ability to secrete SipA. GFP‐InvB recruitment was detected 100 s after docking and reached a maximum 600 s after docking. The authors coupled this analysis with time‐lapse immunofluorescence on fixed samples where *Salmonella* expressed SipA^M45^ during infection of COS‐7 cells. This confirmed intrabacterial depletion of the SipA pool during this time‐frame. SipA was later shown to be exposed on the surface of the SCV after cellular invasion where it cooperated with SPI2 effectors to promote SCV maturation (Brawn et al., 2007).

Although this assay proved to be sensitive for effectors that localise in foci (100 GFP‐InvB fragments sufficient for detection with 2 molecules bound per molecule of SipA effector) (Schlumberger et al., 2005), more diffusely localised effectors such as SopE could not be monitored in real time. Although translocation can be assessed using wild type bacteria, the technique requires genetic modification of the host cell via transfection, thereby limiting the assay to easily transfected cell lines. It is also important to note that while the authors detected translocation as early as 16 s after docking, the kinetics of such measurements are indirect as they rely upon the recruitment of GFP‐InvB to the site of effector localisation. The technique also requires in‐depth knowledge of effector protein‐chaperone pairings, a factor which has limited the applicability of the method for other bacterial species/effectors.

### 2.5 Direct effector labelling with tetracysteine‐FlAsH

Another method that allows for rapid and sensitive detection of effector protein translocation is the tetracysteine‐fluorescein biarsenical hairpin binder (4Cys‐FlAsH) tagging approach. This system requires the fusion of a 12/18‐residue tag containing a 4Cys hairpin to the effector of interest. The effector can then be detected by staining with FlAsH dye, which only becomes fluorescent after binding to the 4Cys peptide tag (Hoffmann et al., 2010). This approach has been used to directly label *Shigella* effector proteins IpaB and IpaC and monitor their localisation in both fixed bacterial and host cells. The effectors were found to be diffusely scattered in the bacterial cytosol prior to secretion and after injection, localised to the actin‐rich membrane ruffles underlying invading bacteria (Enninga, Mounier, Sansonetti, & Van Nhieu, 2005). The approach proved particularly useful for monitoring real‐time translocation of IpaB and IpaC into the host cell. Four‐dimensional spinning disc confocal microscopy revealed immediate depletion of the intrabacterial pool of IpaB and IpaC with half‐maximal secretion being observed at 4‐min postdocking. While depletion within the comparatively small bacterial cytosolic milieu could be quantified in real time, real‐time localisation within host cells required modification of the technique. It was observed that the incorporation of a 3×4Cys tag improved affinity for FlAsH dye, leading to improved signal intensity. This allowed for chromosomal expression of *Salmonella sopE2* and *sptP* fusions from their native promoters and for detection of labelled effectors in host cells (Van Engelenburg & Palmer, 2008).

Advantages of the 4Cys‐FlAsH system include the relatively small size of the peptide tag (12 amino acids) and the FlAsH dye (0.7 kDa), which allow for minimal impact on functionality and secretion efficiency. A red fluorescent variant of the FlAsH dye–resorufin arsenical hairpin binder (ReAsH) allows for flexibility of labelling (Crivat & Taraska, 2012). Importantly, both FlAsH‐EDT_2_ and ReAsH‐EDT_2_ exhibit minimal fluorescence when unbound to their peptide ligands, as such, unlike the fluorescent chaperone‐based detection system, background fluorescence levels from unbound reporter molecules are not problematic. Biarsenical dyes have been shown to have toxic effects on eukaryotic cells (Gaietta et al., 2002). As such, this labelling system is inherently better suited to the study of rapidly translocated effectors (<1‐h post infection). Although FlAsH concentrations of up to 20 μM have been shown to have negligible effects on bacterial growth and viability, increasing dye concentration did cause a dose‐dependent decrease in actin foci formation and bacterial internalisation (important Shigella T3SS phenotypes) (Enninga et al., 2005). Because higher dye concentrations can improve signal from labelled effectors, it has been suggested that the dye concentration/interference with functionality (either direct or indirect) must be carefully balanced for each effector fusion being studied. Another important consideration is that while low signal intensities can be overcome using 3×4Cys tags, thus allowing endogenous expression and real‐time microscopy, the larger (42 residue) tag may result in functional interference with specific effectors, although this was not the case for *Salmonella* SopE2 or SptP (Van Engelenburg & Palmer, 2008).

### 2.6 Fluorescence complementation via split‐GFP

Fusion of GFP to proteins can induce mis‐folding and also prevents effector protein transport through the T3SS needle complex (Akeda & Galán, 2005; Cabantous, Terwilliger, & Waldo, 2005). As such, translocation and subcellular localisation could not be studied using traditional approaches. Although transfection allows for expression of effector‐GFP fusions in host cells, effectors may function improperly when translocated in isolation, and it has recently been demonstrated that localisation of host‐expressed effectors can be different to that of effectors translocated via the injectisome (Van Engelenburg & Palmer, 2010). Split fluorescent protein tagging has been developed as a means of reducing mis‐folding and functional perturbations associated with canonical GFP labelling. After extensive investigation of split‐GFP fragments for fluorescence complementation, it was observed that fluorescence could be restored to GFP by fusion of the eleventh strand of the GFP beta barrel (GFP_11_) to a protein of interest and reassociation with GFP_1–10_ within the cell (Cabantous et al., 2005). Minimal mis‐folding was observed with the GFP_1–10_/GFP_11_ fragments compared with other previously reported split‐GFP approaches.

At only 18 amino acids in length, the GFP_11_ tag was unlikely to have detrimental effects on Type 3 effector function or translocation. The GFP_1–10_ fragment could be expressed from a plasmid transfected into the host cell. This system was applied to the *Salmonella* SPI2 effector proteins PipB2 and SteA, revealing spatial segregation of the effectors with respect to tubular dynamics of the SCV (Van Engelenburg & Palmer, 2010). PipB2 was found to be associated with endocytic and trans‐golgi tubules, whereas SteA was exclusively associated with endocytic tubules with both effectors tightly regulating SCV positioning and maturation. Lateral movement of PipB2 along tubules was demonstrated using fluorescence recovery after photobleaching microscopy. Importantly, the authors also demonstrated that ectopic host expression of PipB2‐GFP resulted in accumulation of the effector at the cell periphery, whereas T3SS translocated PipB2‐GFP_11_ was localised to the tubular network, thereby highlighting the importance of expressing tagged effectors in the bacterial cell rather than transfected host cells.

In recent years, many advances have been made in split‐GFP labelling technologies. In an analogous manner to the use of a trimeric 4Cys fusion tag, after fluorescence complementation a tandem repeat β‐tubulin‐GFP_11×7_ fusion was found to exhibit equal signal intensity at seven times lower exposure rates and with nine times slower photobleaching than the single β‐tubulin‐GFP_11_ fusion (Kamiyama et al., 2016). Interestingly, a recent study demonstrated that 3×GFP_11_ labelling of the *Salmonella* SPI2 effector proteins SseF, SseG, and SlrP resulted in higher fluorescence intensity; however, due to low levels of expression from their endogenous promoters, an experimental set‐up involving plasmid‐based expression driven by the stronger *steA* promoter was favoured over chromosomal tagging. The resultant signal amplification allowed for real‐time identification of differential effector localisation in infected primary macrophages and HeLa cells (Young, Minson, McQuate, & Palmer, 2017). A tripartite split‐GFP labelling method has also been developed to allow the study of protein–protein interactions and to reduce background levels due to aggregation and spontaneous reassociation that are sometimes observed with the GFP_1–10_/GFP_11_ system. The tripartite system involves fusion of the small GFP_10_ and GFP_11_ β‐barrel strands to two distinct interacting proteins. Interaction can be detected by induction of GFP_1–9_ expression within the host cell. Tripartite GFP complementation resulted in lower background fluorescence than bimolecular fluorescence complementation (Cabantous et al., 2013). Application of this system for the study of effector proteins could expand on spatial knowledge by allowing for confirmation of molecular interactions within the host cell, while also reducing background levels due to aggregation of the larger GFP_1–10_ fragment. Another interesting advance has occurred in the field of plant pathology where transgenic *Arabidopsis* lines have been developed, which express GFP_1–10_ targeted to specific subcellular localisations. These transgenic lines successfully allowed identification of segregation of the *Pseudomonas syringae* effectors AvrB (plasma membrane foci, with fluorescence complementation at 3 hpi) and AvrRps4 (cytoplasm and nucleus, with fluorescence complementation at 6 hpi) (Park, Lee, Woo, Choi, & Dinesh‐Kumar, 2017).

Although split‐GFP labelling can be used in real time live cell imaging, it is important to consider that fluorescence complementation depends upon the kinetics of reassociation between the constitutive fragments of GFP, which can take 15 to 30 min (Rodrigues & Enninga, 2010). In the case of PipB2‐GFP_11_, fluorescence complementation was first detected 4‐h post infection, whereas effector translocation can be detected by Western blotting 2‐h post infection (Van Engelenburg & Palmer, 2010). As such, the system is poorly suited to analysis of rapidly injected effectors such as those secreted by *Salmonella* SPI1. Another obvious disadvantage of the system lies in the fact that both the pathogen and the host must be genetically modified. Typically, the host cell is transfected with a plasmid carrying the GFP_1–10_ fragment, and as such, the system is primarily suited to cell lines that can be readily transfected.

### 2.7 Direct effector labelling with light, oxygen and voltage‐sensing domain

Light, oxygen and voltage‐sensing (LOV) domains are found in a variety of photoreceptors in bacteria, fungi, and plants (Christie, 2007). These domains bind the endogenous flavin mononucleotide chromophore, yielding fluorescence properties with spectral similarity to GFP. Advantages of the LOV domain over GFP (≈25 kDa) include its smaller size (≈10 kDa), stability in a wider pH and temperature range, stability in anaerobic environments, more rapid fluorophore maturation, and photo‐switchable properties, which enable use in super‐resolution microscopy (Buckley, Petersen, Roe, Douce, & Christie, 2015). Given the variable nature of effector protein functionality and the diversity of subcellular target locations already described, stability in a wide variety of conditions is a highly desirable property for an effector protein detection tag. The LOV domain from Arabidopsis thaliana has undergone extensive manipulation to improve its applicability for various fluorescence‐based approaches. phiLOV—a brighter and more stable fluorophore with enhanced recovery after bleaching—was obtained through molecular evolution and structural tuning (Chapman et al., 2008; Christie et al., 2012). To improve expression and resultant fluorescence intensity from bacterial expression constructs, codon usage of the iLOV domain was optimised for E. coli to produce phiLOV2.1 (Gawthorne et al., 2016).

Fusion of this optimised LOV domain to EHEC Tir did not interfere with effector translocation or functionality, and Tir‐phiLOV could be readily visualised both in bacterial cells prior to secretion and within A/E lesions on the host cell after bacterial docking (Gawthorne et al., 2016). Translocation of Tir‐phiLOV was successfully monitored by real‐time live cell imaging with depletion from individual bacterial cells and accumulation in the host cell in typical actin‐rich pedestals between 90‐ and 140‐min post infection. The authors did however observe a large degree of heterogeneity with respect to inter‐bacterial dynamics of secretion. Importantly, phiLOV when expressed without an effector protein fusion was retained within the bacterial cell, thus serving as an appropriate negative control for effector‐dependent translocation. In the same study, the authors could demonstrate polar localisation of *Shigella* IpaB‐phiLOV prior to translocation, followed by complete depletion from the bacterial cell and accumulation at entry foci between 15‐ and 45‐min post infection. There was no significant difference in invasion between the *Shigella* wild type and the IpaB‐phiLOV expressing strain indicating minimal functional interference.

The phiLOV fusion has also recently been used in the study of the *Salmonella* SPI1 effector SipA. During infection in an *ex vivo* ileal loop model, SipA‐phiLOV was found to colocalise with activated caspase 3 at villus tips when visualised by multiphoton microscopy (McIntosh et al., 2017). The successful tracking of effector‐phiLOV fusions in intact organs indicates a strong likelihood of applicability to in vivo infection models.

Advantages of LOV domain fusions include the lack of a requirement for addition of harsh/toxic fluorophores such as FlAsH due to the ability of LOV to bind flavin from within the cell. Unlike split‐GFP and fluorescent chaperone‐binding approaches, the effectors are directly labelled, so translocation kinetics are independent of fluorophore recruitment or maturation. Fluorescence is obtained without genetic modification/overexpression of any host cell components thereby increasing experimental flexibility. Furthermore, as the effectors are directly tagged, background levels are less problematic. Spatial resolution at the nanometre scale can be obtained by super‐resolution microscopy and a technique known as correlative light electron microscopy (CLEM). In CLEM LOV‐tagged proteins can be localised not only by their fluorescence properties (in real time) but also by their ability to photooxidise diaminobenzidine (in ultrathin transmission electron microscopy sections) (Shu et al., 2011). The correlated analysis allows for rapid, sensitive localisation at extremely high resolution. CLEM represents a particularly attractive, and as yet unexplored avenue for research in effector protein localisation.

As with many of the approaches described herein, fluorescent signal appears to be the major challenge. While considerable advantages exist over GFP, fluorescence intensity of optimised Tir‐phiLOV2.1 was still three‐fold lower than that of Tir‐GFP (Gawthorne et al., 2016). As a result, Gawthorne *et al*. (2016) focused attention on Tir and IpaB, which are highly expressed and accumulate in discrete locations in the host cell after translocation. EHEC Map was not further investigated due to low fluorescence levels, presumably resulting from lower expression than Tir. It would be interesting to assess whether tandem repeats of phiLOV or expression from an inducible promoter would result in higher signal and allow for the study of Map‐phiLOV localisation in host cells.

### 2.8 Single molecule super‐resolution nanoscopy

A recent study has described the use of 2D and 3D single‐molecule switching super‐resolution microscopy to analyse the distribution of *Salmonella* Type 3 secretion systems, their sorting platform components, and the effector protein SopB in living bacterial cells (Zhang, Lara‐Tejero, Bewersdorf, & Galán, 2017). Prior to secretion, SopB‐mEos3.2 fusions were found to localise to distinct clusters within the bacterial cytosol independent of the secretion apparatus or sorting platform. This has important implications for our understanding of how Type 3 secretion systems interacts with their cognate effectors and highlights the utility of super‐resolution single molecule techniques for the study of effector localisation. Although inherently more challenging, it will be interesting to observe if such techniques can be employed to analyse the distribution of effector proteins in host cells following translocation.

The self‐labelling enzymatic SNAP and Halo tags have also recently been used with super‐resolution‐compatible fluorophores to analyse localisation of InvC and SpaS, which are inner membrane complex components of the *Salmonella* SPI1 T3SS (Barlag et al., 2016). Although this study did not involve the analysis of effector localisation, the technique has potential for application in the analysis of single molecule super‐resolution localisation of T3SS effector proteins. Interestingly, SNAP and Halo tags can be labelled with tetramethylrhodamine, which has previously been used for CLEM (Liss, Barlag, Nietschke, & Hensel, 2015); a factor which expands the repertoire of applications for these reporters.

## 3 CONCLUDING REMARKS

It is worth noting that most effector‐tracking studies have focused on highly expressed effectors that are concentrated within specific sites in the host cell. These include Tir, IpaB, IpaC, SipA, PipB2, and SteA. Poorly expressed or diffusely localised effectors present a greater challenge for microscopic tracking, particularly in real time where photobleaching is a concern. The application of tandem repeat fluorescent tags is sometimes not sufficient to overcome issues with low signal from such effectors (Park et al., 2017; Young et al., 2017). Multimerisation of tags comes at the cost of increased propensity for aggregate formation and an increased risk of interfering with functionality. Many fluorescent tagging approaches have employed plasmid‐based expression and driven expression from stronger heterologous promoters in attempts to improve signal levels. Such efforts may result in experimental artefacts due to antibiotic selection and improper concentrations of the over‐expressed effector in the host cell. The use of brighter fluorescent proteins and more sensitive microscopy technologies would be extremely beneficial for real‐time tracking of truly endogenously expressed chromosomal fusions. The SunTag with its capability to bind 24 molecules of GFP represents an attractive option for tracking effectors with lower levels of expression (Tanenbaum, Gilbert, Qi, Weissman, & Vale, 2014). Modern microscopy methods such as spinning disc confocal microscopy, multiphoton laser scanning microscopy, correlative light and electron microscopy, and single‐molecule switching super‐resolution microscopy can be employed to minimise photobleaching, assist with focusing on dynamic samples (such as those containing actively invading bacteria) and gain spatial information at much‐improved resolution. Although some of the technologies described herein are suitable for tracking rapidly translocated effectors (4Cys‐FlAsH and phiLOV), others are complicated by delays in fluorophore recruitment/maturation (GFP‐InvB and split‐GFP) and as such are primarily suited to analysis of effectors that are translocated at later time‐points. With so many available technologies, careful consideration should be given to the suitability of both the effector fusion tag and microscopic analysis method used to ensure optimal results.

## CONFLICT OF INTEREST

The authors declare no conflicts of interests.
